# How Can Resource-Exhausted Cities Get Out of “The Valley of Death”? An Evaluation Index System and Obstacle Degree Analysis of Green Sustainable Development

**DOI:** 10.3390/ijerph192416976

**Published:** 2022-12-17

**Authors:** Xinyu Zhuang, Xin Li, Yisong Xu

**Affiliations:** 1 College of Quality & Standardization, Qingdao University, Qingdao 266071, China; 2 School of Tourism and Geography Science, Qingdao University, Qingdao 266071, China; 3 Advanced Institute of Culture & Tourism, Qingdao University, Qingdao 266071, China; 4 School of Business, Qingdao University, Qingdao 266071, China

**Keywords:** resource-exhausted cities and urban transformation, evaluation models, obstacle degree analysis, green development, sustainable cities

## Abstract

Resource-based cities are suffering from resource scarcity and environmental deterioration. Spirit, vitality and prosperity are disappearing and cities have moved towards “the valley of death” in terms of urban development. This typically appears in environments where it is difficult to maintain sustainable development. Based on empirical analysis, a qualitative analysis method for the selection of evaluation indicators, as well as a quantitative analysis method for index weighting and principal component extraction for constructing a three-level evaluation index system of green development for coal-resource-exhausted cities, was adopted. This study also discussed the life cycle at different development stages of resource-based cities, including mature resource-based and growing resource-based cities. We further argued that the obstacle degree can act as an evaluation basis and make recommendations accordingly to improve the green development of cities. Through star-standard divisions and statistical analysis, it can be explicated that the increase in green development in the first stage is greater than that in the later stage, which is more obvious in cities with lower stars. The results also show the evolution trend and stability coefficient. There is no end in sight for urban green development, and this study can provide a new perspective to relieve the declining trend and promote green sustainable development.

## 1. Introduction

In the process of human development, humans have adopted an extensive development model with “high input, high consumption and high pollution” [1,2,3], constantly consuming resources and discharging waste to the environment in order to meet growing material needs and ensure the rapid development of the economy and society [4,5]. After years of resource depletion, large-scale, high-intensity, large-area resource mining and the rapid development of high-energy-consuming industries, many resource-based cities have suffered serious resource loss and environment deterioration [6,7]. However, people’s demand for economic development and social progress has not diminished. Therefore, there is a contradiction between people’s unlimited demand and the limited supply of resources and the environment [8]. The continuous intensification of this contradiction forces resource-based cities to urgently undertake a green transformation and apply an intensive development model based on “low input, low consumption and zero pollution” [9,10]. In order to reduce harm to the environment, reduce carbon emissions and achieve green growth, green circular and sustainable development has become the common goal of urban development on a worldwide scale [11,12,13,14,15]. Consequently, human society has entered a stage of green low-carbon development.

In recent years, the COVID-19 pandemic has also threatened city renewal and energy consumption [16], and energy issues in European countries have had a great impact on the economy and urban development. Northern European countries, for example, are investing heavily in green energy, with wind farms now adorning the coast in many regions, in a race to become carbon neutral and reduce undue reliance upon Russian gas. Hence, a large number of resource-based cities have actively undergone a green transformation [17] in terms of their resource base, aiming for low consumption and zero pollution. Among them, there are cities showing good transformation results; however, there are also cities with unsatisfactory transformation [18,19], even exhibiting high unemployment and loss of technical talents in resource-based enterprises alongside environmental pollution [20,21]. At present, many cities promoting a green transformation are faced with problems implementing this due to long-term over-exploitation, such as resource depletion, slow economic growth and the governance of the ecological environment [22,23,24]. In order to solve the above problems, it is urgent that alternative industries are found to transform the economic structure so that the economy can achieve long-term, stable and green development.

China is a country with a large amount of coal resources compared with the rest of the world. The resource-based cities that rely on coal resource mining and primary processing account for one-third of the cities in China, and they have had a great effect on social and economic development in the past [18,24]. However, under the influence of industrial development and non-renewability in the process of coal resource development, in the future, coal-led industries will decline in clusters [22], which will not only lead to a decline in industrial benefits and the shrinkage of resource-led industries, but also lead to economic recession and increasingly serious environmental and ecological problems, because alternative industries have not yet been formed [24,25]. Hence, realizing the green transformation and development of a resource-exhausted city has become one of the main problems to be solved to improve the ecological environment and sustainability [26,27].

To date, scholars have carried out much research on the green transformation of cities, but there is still a lack of studies on this topic to date. First, based on the characteristics of typical coal-resource-exhausted cities, which are distributed in different regions and have different regional characteristics, this can reflect the impact of regional differences on urban development to a certain extent, which has rarely been mentioned in other studies. Second, stage characteristics of the evolution and life cycle of coal-resource-exhausted cities are discussed in this study, whereas few have considered this in previous research. Third, the coordination between qualitative analysis and quantitative analysis methods adopted in this study can show the research results more deeply. This study attempts to obtain the contribution rate, cumulative contribution rate and principal component load by calculation, which have been rarely mentioned previously. Furthermore, although some prior research has focused on the evaluation index system of green development, we further analyzed the obstacle factors and obstacle degree of green development on resource-exhausted cities by empirical analysis.

Hence, in order to thoroughly understand the green development and transformation of resource-exhausted cities, in our preliminary study, we evaluated the relevant literature to determine whether they applied to resource-based cities, resource-exhausted cities, economic transformation, green transformation, green development, etc., and summarized the specific research direction. According to the distribution of resource-exhausted cities in three different years (2008, 2009 and 2011), and the planning document (GF [2013] No. 45) issued by the State Council on 12 November 2013, a total of 33 cities in China were defined as coal-resource-exhausted cities and selected for field investigation as the typical cases, which lasted for two years, to obtain data on green development. Furthermore, we also discussed the issues with experts to collate their suggestions regarding the green development of resource-exhausted cities. We further discussed the life cycle and development stages of resource-exhausted cities, then analyzed and constructed the evaluation index system and obstacle degree of green development. It was expected that this would allow us to explore green development suitable for coal-resource-exhausted cities on the basis of understanding the situation concerning economic and social development versus ecological and environmental protection.

This paper is organized as follows: Section 2 reviews the literatures, theories and research methods regarding city transformation and green development, and highlights the problems and aims of this study. In Section 3, the selection of coal-resource-exhausted cities, evaluation index of green development and the weight determination of the evaluation index of green development are proposed, while Section 4 presents the evaluation model and improved obstacle degree model of green development, evaluates the index system of the green development of coal-resource-exhausted cities and proposes factors acting as obstacles to green development. Findings, theoretical contributions, recommendations and future directions are provided in Section 5. Conclusions are summarized in Section 6.

## 2. Literature Review

### 2.1. Resource-Exhausted Cities and Green Transformation

According to the level of green development, resource-based cities can be divided into three types, that is, mature resource-based cities, growing resource-based cities and resource-exhausted cities. Resource-exhausted cities refer to cities in which the development of mineral resources has entered the process of decline or depletion [28,29]. Therefore, some researchers have defined this type of resource-based cities “resource-declining cities” [30]. Generally, a city whose cumulative extraction reserves have reached more than 70% of the original measured amount or that can only maintain mining for five years at the current technical level and mining capacity can also be called a “resource-exhausted city” [31,32,33]. Due to the rules of the development of resource industries and resource-based cities, resource-based cities are bound to experience the life-cycle processes of construction, prosperity, recession, transformation, revitalization or extinction, and they are faced with the problem of how to maintain sustainable development, extend life-cycle engineering [34] and maximize the lifetime of the city. Resource-exhausted cities have four common characteristics: First, with the depletion of resources, industrial benefits decline; second, the industrial structure is single, the resource industry is shrinking and the alternative industry has not yet formed; third, the total economy is insufficient and local financial resources are weak; fourth, the income of a large number of employees is lower than the per capita level of urban residents.

Green transformation refers to the transformation of the development model to sustainable development with the construction of the ecological environment as the guide [35], circular economy as the foundation and green management as the guarantee, so as to realize resource conservation, environmental protection, ecological balance and the harmonious development of humans, nature and society [36,37]. At the core is transformation from the traditional development model to the scientific development one [38], which is also a kind of development pattern of the deviation and the separation of the economy, society and ecology for the harmonious coexistence between humans and nature, so as to achieve the coordinated development of the economy, society and ecology [39,40,41,42,43,44,45].

The transformation of resource-exhausted cities is also a prominent phenomenon experienced or being experienced in the economic and social development of all countries [35,46]. Not only in China, but also in the world, many cities are facing transformation problems, which require a long-term historical evolution. With the depletion of resources, resource-based cities are also faced with serious historical problems in the process of development. The imbalance between resources, social and economic development, urban construction and ecological environment protection has led to the decline of cities. From the Rust Belt in the United States to the Ruhr District in Germany, from Lorraine in France to Kitakyushu in Japan, from Bolivar in Venezuela to Baku in Azerbaijan, the sinking and re-starting of resource-exhausted cities have been constantly alternating.

In the early and middle stages of industrialization, it had already been a representative problem. Europe, the United States and Japan have had very developed mining industries in the modern period, which also created a developed mining economy and formed an entire legal system and civilization, such as the Ruhr mining area in Germany and Lorraine mining area in France [47], both of which were once famous towns of coal and iron [48]. In the process of industrialization, the mining industries in Europe [47] and Japan [49] have gradually declined. However, the mining cities in these countries did not decline with the demise of the mining industry, they reasonably solved the historical problems of mining cities through national policy guidance and industrial structure optimization. They successfully carried out economic transformation, removed the shackles of excessive dependence on domestic and regional mineral resources, and revived the traditional mining areas to achieve regional economic balance, coordination and sustainable development [50]. The transformation and sustainable development of resource-exhausted cities have been successfully achieved (for example, Germany, France and Japan), and some cities have achieved this reasonably well (for example, Italy, Greece, Spain and the United States); research and case studies can provide valuable experience and reference for better solving the problems of resource-based cities (Table 1).

From Table 1, it can be seen that the transformation modes of resource-based cities under different society and economic systems are different, and the choice of transformation mode is often rooted in a deeper urban development. Throughout the transformation practices all over the world, the policy orientation can be divided into four categories (Table 2).

After a few decades of development, China’s resource-based cities are gradually being threatened by resource depletion [51]. Similar to non-renewable resources, natural resources are decreasing year by year [52,53,54]. Only the exploitation of resources has been considered while ignoring the conditions and factors of urban development, which has resulted in many problems. Resource-based cities in China are facing complex and diverse difficulties [55]. In 2013, 69 of the 262 resource-based cities published in the Sustainable Development Plan of China for resource-based cities (2013–2020) were in a recession. At present, with the countdown to China’s carbon emission peak, there is little left in the transition period for resource-based cities.

### 2.2. Green Development

Green development is regarded as an important part of scientific development; the concept of green development converges with the essence of an “ecological civilization” [45,55], “green economy” [56] and “harmonious society” [57]. Green development is not only an idea but also a strategic decision. Hence, this study reviewed the previous research, and analyzed and constructed the evaluation index system of green development for coal-resource-exhausted cities. It is expected that the green development suitable for coal-resource-exhausted cities will be explored on the basis of realizing the situation of economic and social development versus ecological and environmental protection.

Researchers have a more realistic interpretation of green development. In the 1960s, G. Hardin used mathematical models to analyze the relationship between environment and economic development and highlighted that one of the ways to avoid the tragedy of the Commons is to promote green development [58]. In 1989, D. Pearce, a British environmental economist, mentioned that the core of green development is to protect resources and the environment as well as to maintain ecosystems in “*Blueprint for a green economy*” [59,60]. Combined with research regarding cities, green development is to implement the green concept, including the demand concept, resource concept, environmental concept and benefit concept, so as to promote the sustainable development of society and ecology [61].

On the other hand, green development, in the modern sense, is the upgrading of sustainable development. The essence of green development is thought to protect the ecological environment and realize sustainable development under the constraints of the resource carrying capacity and ecological environment capacity [62]. Green development is also proposed as the second generation of sustainable development, and humanism is the core of sustainable development [63]. It not only represents the contradiction between the population, resources and the environment in the traditional concept of development, but also the overall crisis between humans and the climate.

### 2.3. Comparision of the Literature on Urban Transformation and Sustainable Development

Below, we have collated the literature on the green transformation and sustainable development of cities in detail, which indicates differences among research methods and conclusions (Table 3).

“Lucid waters and lush mountains are invaluable assets” is the strategic direction of green development and ecological and environmental protection in China [72,73]. Its essence is to promote the life cycle and achieve sustainable development [74,75]. The traditional development mode of high energy consumption and high pollution is no longer applicable to the development concept and needs. The green and ecological development mode can minimize the pressure caused by economic activities on the environment so as to achieve green development [76]. Hence, on the basis of the characteristics of 33 coal-resource-exhausted cities in different regions, this study established a three-level indicator system for the green development of coal-resource-exhausted cities by classifying the star-level standard of the green development score and conducting statistical analysis from 2005 to 2015. The results can provide a reference for the green transformation of resource-exhausted cities. In addition, on the basis of analyzing the development characteristics of coal-resource-exhausted cities, the principal component extraction method was adopted to build the evaluation index system of green development, and the obstacle degree model was used to find the methods of green development that can provide a decision-making basis for urban transformation and the ecological environment, and solve the issues surrounding the green sustainable development of cities.

## 3. Research Design

### 3.1. Data Collection

#### 3.1.1. Selection of Coal-Resource-Exhausted Cities

**Figure 1:**
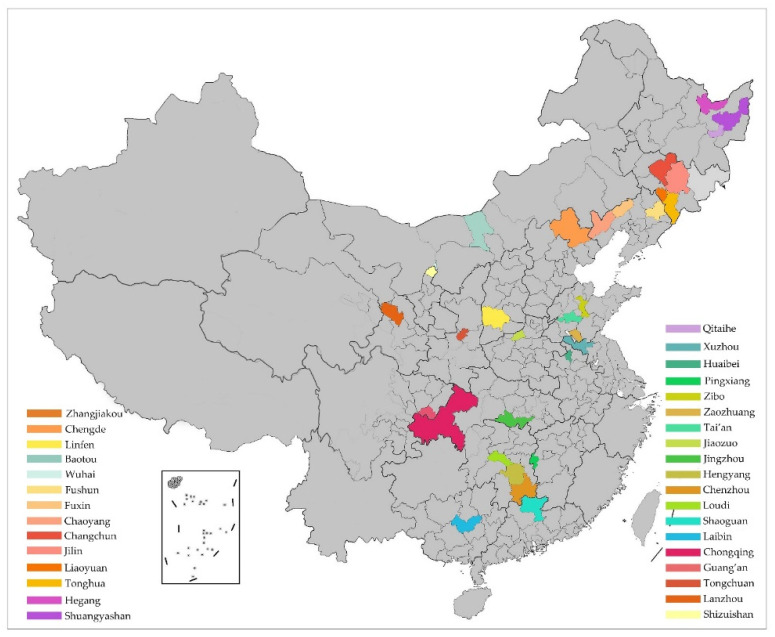
Location and distribution of the object cities. Source: Author self-painting.

In 2013, the State Council of PRC issued the document [2013] No.45 and established 33 coal-resource-exhausted cities, accounting for more than half of the total number of cities with declining resources; this shows that the development of coal-resource-based cities is not sustainable. According to the list of resource-exhausted cities published by the National Development and Reform Commission in 2008, 2009 and 2011, China Statistical Yearbook (2020) [77], we selected the prefecture level, and cities defined as coal-resource-exhausted were included as the research cases in this study. Specifically, we included: Zhangjiakou, Chengde, Linfen, Baotou, Wuhai, Fushun, Fuxin, Chaoyang, Changchun, Jilin, Liaoyuan, Tonghua, Hegang, Shuangyashan, Qitaihe, Xuzhou, Huaibei, Pingxiang, Zibo, Zaozhuang, Tai’an, Jiaozuo, Jingzhou, Hengyang, Chenzhou, Loudi, Shaoguan, Laibin, Chongqing, Guang’an, Tongchuan, Lanzhou and Shizuishan, totaling 33 cities, as shown in Figure 1. Below are the location and distribution of the object cities. 

The 33 coal-resource-exhausted cities relate to seven geographical regions in China. Influenced by history, mining technology and other developmental factors, they are mainly concentrated in Northeast China (10 cities), East China (6 cities), North China (5 cities), Central China (5 cities), South China (1 city), Southwest China (3 cities) and Northwest China (3 cities), involving 19 provinces. Urban planning departments generally classify cities according to the total population number (including the non-agricultural population in urban centers and suburban areas), and cities can be divided into four levels in size: small cities, medium-sized cities, large cities and mega cities. According to the standard of city size classification in China, there are 3 mega cities, 5 big cities, 6 medium-sized cities and 19 small cities in terms of the coal-resource-exhausted cities in China (Table 4).

#### 3.1.2. Selection of Evaluation Index on Green Development

Green development emphasizes the rules of nature, maintaining the ecological environment and developing green ecological industries. It aims to build an industrial chain of resources, the environment, products, regeneration, utilization and recycling, and realize the coordination and unity between man and nature, economy and society [64,65]. Therefore, when constructing the evaluation index system of green development for coal-resource-exhausted cities, the selected indicators should be representative and comprehensive and fully reflect all aspects of green development in such cities. On the basis of the principles of consistency and being systematic, typicality and comparability, and feasibility and scientificity, combined with the current situation of green development in China, this study selected three-level criteria indicators to build the evaluation index system of green development for coal-resource-exhausted cities.

With regard to the selection, based on the National Sustainable Development Plan for Resource-based Cities (2013–2020) (GF [2013] No. 45) issued by the State Council and the China Statistical Yearbook of Cities in 2020 [77], the urban sustainable development theory, the social ecological systems theory [68], the industrial structure evolvement theory [30], the evolutionary resilience theory [18] and the problems found in the field survey, we selected typical representative indicators. The reasons are as follows: First, resources and the environment. Considering the situation of green development, environmental indicators are essential, and resource issues are also the focus of its consideration as it is a coal-resource-based city [24]. The second reason is economic development. Coal-resource-exhausted cities are developed based on coal resources, and urban economic development almost depends on coal [64]. Once the coal resources are exhausted, the cities will inevitably be faced with new pillar industries to support urban economic development. The third reason is population and social welfare. Coal-resource-exhausted cities have a large number of low-skilled personnel who have migrated [66,77]. Once exhausted, there will be a large number of laid-off workers who have no work and live off of government welfare. Consequently, the specific indicators chosen were as follows:

(1) Resources and the environment. The resources and the environment are the material basis for human survival and societal development. This indicator mainly reflects the field of the ecological environment, paying attention to the impact of economic development. In particular, this includes the impact of industrial discharge on the quality of the ecological environment, the impact of waste and wastewater treatment in daily life on the urban environment and the impact of urban greening on improving the quality of the living environment for urban residents [62].
(2) Economic development. Economic development is accompanied by the growth of the economic aggregate, and the increase in the economic aggregate can drive an increase in people’s income. As the economy increases, it will drive changes in economic and financial structures. Therefore, the measurement of economic development mainly includes four aspects: the growth of regional GDP, the improvement of income, the investment of science and education and the optimization of economic structure [14,77].
(3) Population and social welfare. The population in the evaluation index system refers to the population living in cities and market towns. Social welfare mainly focuses on human development and conducts an all-round investigation from the perspectives of transportation, education, medical treatment, etc. [64]. The investigation of green development can be realized through the green degree of products and services; furthermore, the development of human beings can benefit from a green economy.

On the basis of selection of the criteria-level indicators, the indicators needed to be extended to the index layer. Therefore, according to the actual situation of coal-resource-exhausted cities and following the design principles of the evaluation index system of urban green development, 23 secondary indicators were selected, corresponding to three evaluation criteria. The secondary indicators selected in this study have the following characteristics: First is the consistency between secondary indicators and primary indicators. The secondary indicators need to be selected based on the requirements of the primary indicators, that is, the criteria layer, which can reflect the purpose of the primary indicators and is closely related to the content of the primary indicators. Second is the pertinence of secondary indicators to specific cities. Coal-resource-based cities have their unique urban structure and judgment standards; thus, while selecting the secondary indicators, the pertinence of specific urban types should be focused on so that the indicators can more effectively reflect the actual situation of target cities. Third is the operability of data collection of indicators samples. The secondary indicator is the most specific indicator layer in this study, which needs to be weighted by objective methods. Therefore, the selection of secondary indicators must be supported by corresponding sample data. Fourth is the proportional treatment of secondary indicators. When obtaining sample data, there are various unit forms and the scale of each sample is different, which cannot simply be compared directly. Therefore, in the preliminary data process, the sample data were scaled to convert them into a proportional form so as to partially eliminate the influence on data units and city scale. In view of the above requirements and characteristics, the index layer of this study was able to convey the purpose of the criterion layer according to the specific situation of coal-resource-exhausted cities and provide operable sample data for the objective weighting of indicators. The specific index system is shown in Table 5.

The reason for the green transformation of resource-based cities is the result of the systematic operation of three factors, namely resources and the environment, economic development, and population and social welfare. Internal contradictions of the four factors constitute the direct driving force of the green transformation of resource-based cities. Therefore, evaluating the green development from these four factors is helpful to relieve the contradiction between the unlimited demand in economic and social development and the limited supply in terms of the operation of resources and the environment. We also analyzed the intensification and mitigation of the contradiction. The specific methods of realizing the green development of resource-based cities are via the green transformation of enterprises, industries and governments, which are directly related to the green transformation effect of resource-based cities at the microcosmic, middle and macroscopic levels. To build a green city is to create a low-carbon development paradigm that runs the concept of green development through the whole process of construction, and takes green production as the basis, green life as the driving force, green ecology as the guarantee and green life as the vision to realize the development paradigm that is suitable for industry, living, entertainment and tourism.

### 3.2. Determination of the Evaluation Index Weight of Green Development

Determining the weight is the second most important element in constructing an evaluation index system of the green development of coal-resource-exhausted cities. In order to ensure the objectivity of the weight determination process, this study used the principal component analysis method to calculate the weight of 23 indicators describing the green development of coal-resource-exhausted cities.

Principal component analysis (PCA) is a mathematical transformation method that simplifies the complex relationship between variables [78]. While avoiding the loss of information as much as possible, the high-dimensional index vector is reduced to simplify multivariable panel data. Then, the principal component coefficient, extracted after dimensionality reduction, is transformed into a weight that can be used as the index weight through mathematical calculation [79].

The advantages of using principal component analysis to process multivariable panel data are as follows: First, it can remove the influence of the correlation of the original indicators on the information repetition caused by the results, so as to ensure the objectivity of the results. Second, the weight of each index produced by the principal component analysis in the operation process is completely based on objective information, which is different from the analytic hierarchy process and can avoid the bias and error caused by subjective factors. Third, it can reduce the workload on the premise of retaining most information as much as possible.

(1) Samples and data sources

This study took 33 coal-resource-exhausted cities identified in the No. 45 Document of the State Council [2013] as samples, and selected the China Statistical Yearbook [77] from 2005 to 2015 as the original data. In order to ensure the feasibility of the operation process, this study used the replacement method on missing values.

(2) Dimensionless data processing

When using principal component analysis, the dimensionless processing of the original data and the impact of positive and negative indicators on the calculation results were focused on. Due to the differences in the index units of the selected evaluation indicators, that is, the data having a dimensional effect, the selected data should be processed as dimensionless. The commonly used dimensionless processing methods mainly include standardization, extremum, etc. However, since the data after standardization can only reflect the impact between indicators and cannot reflect the differences in the degree of variation between indicators, compared with extremum processing methods, it is not suitable for dimensionless processing among multiple variables. Therefore, this study selected the extremum method to process the data as dimensionless.

Since the principal component analysis needs to be unified into positive indicators, the negative indicators in the original indicators should be handled separately. In this study, the negative indicators of the green development of coal-resource-exhausted cities, namely the emission of industrial dust of GRP per CNY 100 million, the emission of industrial sulfur dioxide of GRP per CNY 100 million and the industrial wastewater emission of GRP per CNY 10,000, were dimensionless and treated with Formula (2); the other positive indicators were treated with Formula (1).

The specific formula is as follows:

(1) $$X_{ij}=\frac{\left(x_{ij}-\min x_{i}\right)}{\left(\max x_{i}-\min x_{i}\right)}$$

(2) $$X_{ij}=\frac{\left(\max x_{i}-x_{ij}\right)}{\left(\max x_{i}-\min x_{i}\right)}$$

where $X_{ij}$ is the dimensionless data j of indicator i, $x_{ij}$ is the original data j of indicator i, $\min x_{i}$ is the minimum value of indicator i and $\max x_{i}$ is the maximum value of indicator i.

(3) Data validity test

Before using data samples, it is necessary to test the adequacy of data and samples. The results of the KMO test and Bartlett’s test of sphericity are shown in Table 6.

This study uses Stata 13.0 software to conduct the KMO test, and it was found that KMO = 0.589, which is >0.5; the observed value of Bartlett’s test of sphericity was 10,479 and the corresponding *p*-value was 0, which is far less than 0.05. This shows that the sample data were suitable for principal component analysis.

(4) Calculation of principal component analysis

It is assumed that the data used had several samples, that is, n, and each sample had variables to form an order matrix n×p.

$$X=\left[\begin{array}{cccc} x_{11} & x_{12} & \ldots & x_{1p}\\ x_{21} & x_{22} & \ldots & x_{2p}\\ \vdots & \vdots & \vdots & \vdots \\ x_{n1} & x_{n2} & \ldots & x_{np} \end{array}\right]$$

Calculating the correlation coefficient matrix,

$$R=\left[\begin{array}{cccc} r_{11} & r_{12} & \cdots & r_{1p}\\ r_{21} & r_{22} & \cdots & r_{2p}\\ \vdots & \vdots & \vdots & \vdots \\ r_{p1} & r_{p2} & \cdots & r_{pp} \end{array}\right]$$

$r_{ij}(i,j=1,2,\ldots ,p)$ is the correlation coefficient $x_{i}$ and $x_{j}$ of the original variable, $r_{ij}=r_{ji}$, and the calculation formula is:

(3) $$r_{ij}=\frac{\sum_{k=1}^{n}\left(x_{ki}-{\bar{x}}_{i}\right)\left(x_{kj}-{\bar{x}}_{j}\right)}{\sqrt{\sum_{k=1}^{n}\left(x_{ki}-{\bar{x}}_{i}\right)\sum_{k=1}^{n}{\left(x_{kj}-{\bar{x}}_{j}\right)}^{2}}}$$

We calculated the eigenvalue of the characteristic equation, the eigenvector of the corresponding eigenvalue, principal component contribution rate, cumulative contribution rate and principal component load. The eigenvalue, contribution rate, cumulative contribution rate and principal component load were obtained by calculations, which are shown in Table 7 and Table 8.

It can be seen from the calculation of the eigenvalue and variance contribution rate that the top 12 components contributed 85.8% (more than 85%), that is, the top 12 components could replace 23 original indicators. Therefore, the top 12 components were extracted, namely, principal components 1, 2, 3, 4, 5, 6, 7, 8, 9, 10, 11 and 12.

According to Table 7 and Table 8, the coefficient of each index in the principal component was calculated as follows:

(4) $$\lambda_{ki}=\frac{x_{ki}}{\sqrt{\alpha_{k}}}$$

The normalized weight was calculated according to the coefficient of each index in the principal component, as follows:

(5) $$\beta_{i}=\frac{\sum_{k=1}^{n}\lambda_{ki}\times \rho_{k}}{\sum_{k=1}^{n}\rho_{k}}$$

The normalized weight was:

(6) $$\gamma_{i}^{}=\frac{\left|\beta_{i}\right|}{\sum_{i=1}^{m}\left|\beta_{i}\right|}$$

replacing Equations (4) and (5) into Equation (6) to obtain the normalized weight. There are m indicators, $\gamma_{i}$, which are the normalized weight of the indicator i, $\beta_{i}$ is the non-normalized weight of the indicator i, $\lambda_{ki}$ is the coefficient of the indicator i of the principal component k, $x_{ki}$ is the load value of the indicator i of the principal component k,$\alpha_{k}$ is the characteristic root corresponding to the principal component k,$\beta_{i}$ is the weight of the indicator i, totaling n principal components and $\rho_{k}$ is the contribution rate of the principal component k.

Hence, the normalized weight of each indicator was obtained by substituting the data and the index evaluation system of the green development for coal-resource-exhausted cities was constructed, as is shown in Table 9.

## 4. Research Results

### 4.1. Evaluation Analysis of Green Development

#### 4.1.1. Evaluation Model of Green Development

After obtaining the specific index weight, the evaluation model of green development was further used to evaluate the green development level of 33 coal-resource-exhausted cities from 2005 to 2015. The higher the evaluation index Y of green development, the higher green development level of the city, and vice versa. The specific model is as follows:

(7) $$Y=\sum_{i=1}^{m}\gamma_{i}S_{i}$$

where, Y is the evaluation index of green development, there are m indicators in total, $\gamma_{i}$ is the normalized weight of the indicator i, and $S_{i}$ is the value of the indicator i after standardization.

The score of the green development level of 33 coal-resource-exhausted cities in China from 2005 to 2015 can be obtained by substituting the index weight into Equation (7), as is shown in Table 10.

#### 4.1.2. Evaluation of Green Development of Coal-Resource-Exhausted Cities

Based on the above analysis, we calculated the score and rank of the green development of coal-resource-exhausted cities in 2005, 2010 and 2015 (Table 11).

The specific situation of the green development level of each coal-resource-exhausted city is shown in Table 11. From this table, it can be seen that the top five cities of the green development level were relatively stable. In 2005, the top five cities with the highest level of green development were Lanzhou, Zibo, Xuzhou, Tai’an and Changchun. In 2010, the top five cities with the highest level of green development were Zibo, Baotou, Lanzhou, Changchun and Huaibei. In 2015, the top five cities with the highest level of green development were Zibo, Xuzhou, Lanzhou, Tai’an and Wuhai. Within the frequency range of the cities in the three years [5,15], there were actually eight cities within the coefficient range of [0,1], and the stability index was 0.3, so the rank of cities with a high level of green development was relatively stable.

Zibo was the city with the highest average level of green development and Laibin was the lowest. By calculating the average value of the three years, it was found that the three cities with the highest average level of green development were Zibo, Lanzhou and Xuzhou, reaching 0.56, 0.54 and 0.53, respectively. The three cities with the lowest average level of green development were Laibin, Chaoyang and Chengde, reaching 0.38, 0.39 and 0.41, respectively.

The city demonstrating the fastest change was Wuhai, and the city with the slowest change was Hegang. Over 11 years, the score ranking of green development in Wuhai increased by 23 places, which is 52% higher than that in 2005; Shizuishan’s score ranking of green development also increased by 14 places, including 6 in the first five years and 8 in the next five years; Shaoguan increased by 13 places, characterized by rapid green development in the first five years, rising 11 places, and then rising 2 places in the last five years, which is slightly weak. Hegang, the city with the slowest improvement, fell by 20 places; Qitaihe and Jingzhou also showed slow changes, falling 18 and 13 places, respectively. Although these cities have slowly risen, their level of green development has always improved; however, some cities have regressed in terms of their green development, such as Baotou and Jingzhou, which had a lower level in 2015 than in 2010.

Moreover, we also used the median method to grade the evaluation scores of the green development of coal-resource-exhausted cities in China from 2005 to 2015, divided into four levels: one star, two stars, three stars and four stars. The specific corresponding division and state of the green development level are shown in Table 12.

In order to understand the green development of coal-resource-exhausted cities more intuitively, the data of 2005, 2010 and 2015 were extracted and analyzed.

**Figure 2:**
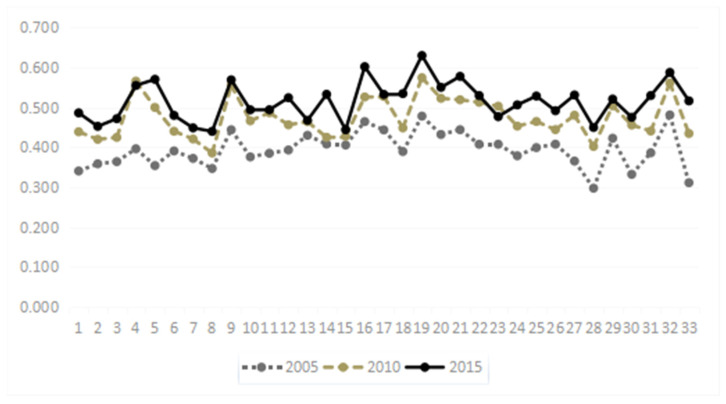
Scatter plot of green development score of coal-resource-exhausted cities in 2005, 2010 and 2015.

As is shown in Figure 2, the ordinate represents the evaluation score of the green development of coal-resource-exhausted cities, the abscissa represents the 1st to 33rd coal-resource-exhausted cities from left to right (in ascending order of zip code), and the three horizontal dotted lines represent the three-star and four-star rating line (0.508), two-star and three-star rating line (0.461) and one- or two-star rating line (0.424), from top to bottom. Referring to Figure 2, the overall situation of the green development level of coal-resource-exhausted cities is clearly as follows:

First, the level of green development of 33 coal-resource-exhausted cities has steadily improved. From the minimum value, the scores in 2005, 2010 and 2015 were 0.297, 0.385 and 0.439, respectively; from the maximum value, the three-year scores were 0.481, 0.574 and 0.629, respectively; from the average score of green development, the three-year average scores were 0.394, 0.474 and 0.514, respectively. Therefore, the level of green development of coal-resource-exhausted cities in China steadily improved from 2005 to 2015.

Second, the increase in green development from 2005 to 2010 grew faster than that from 2010 to 2015. In 2005, 2010 and 2015, 11 years were separated into two stages. Comparing the two-stages of green development, it was found that no city reached a four-star level in 2005 and nine cities reached a four-star level by 2010, while 19 cities were at four-star green development in 2015, accounting for 0%, 27.3% and 57.8% of all coal-resource-exhausted cities, respectively. Furthermore, the number of four-star cities in the second stage increased by 3.2% over the first stage. The number of three-star cities was three, seven and nine in 2005, 2010 and 2015, respectively, while the increased number of four-star cities in the second stage was 3.0% lower than that in the first stage. According to this algorithm, the number of two-star cities in the second stage decreased by 30.2%, which was lower than that in the first stage. The number of one-star cities in the second stage decreased by 78.8% compared with the first stage. Therefore, the overall improvement in the second stage was faster than that in the first stage, and this trend will be more obvious in coal-resource-exhausted cities with a lower number of stars.

### 4.2. Obstacle Degree Analysis of Green Development

#### 4.2.1. Improved Obstacle Degree Model on the Green Development of Cities

The obstacle degree model is a quantitative analysis model to evaluate the specific obstacle factors affecting the measured indicators. The previous obstacle degree model tended to measure the obstacle degree of specific time and location nodes in a single index, but could not measure the comprehensive obstacle degree of a single index. Based on the factor contribution degree and comprehensive deviation degree of the indicators, the obstacle degree model in this study extends the significance of the standardized value of a single index and takes the expectations of all time points and location samples under a single index as the comprehensive value $S_{i}$ after the standardization of a new single index. Consequently, this enables us to determine the obstacle factors affecting the green development of coal-resource-exhausted cities through the obstacle degree model, quantify them and put forward policy suggestions for green development objectively and pertinently. In the improved obstacle degree model, the concepts of factor contribution degree ($W_{j}\times \gamma_{i}$) and index comprehensive deviation degree ($1-S_{i}$) are introduced. The specific formula is as follows:

(8) $$O_{i}=\frac{W_{j}\times \gamma_{i}\times (1-S_{i})}{\sum_{i=1}^{m}W_{j}\times \gamma_{i}\times (1-S_{i})}\times 100\%$$

where, $O_{i}$ represents the obstacle degree of the indicator i to the green development of coal-resource-exhausted cities in a total of m indicators. $W_{j}$ is the weight of the criterion layer j to which the indicator is i. In this study, the value of j is j=1,2,3, $\gamma_{i}$ is the normalized weight of the indicator i and $S_{i}$ is the value of the indicator i after extreme value standardization. At the same time, the obstacle degree of the criterion layer j to the green development of coal-resource-exhausted cities can be summarized by $R_{j}=\sum O_{ij}$.

#### 4.2.2. Analysis of Obstacle Factors on the Green Development of Resource-Exhausted Cities

The obstacle degree of each obstacle factor affecting the green development of 33 coal-resource-exhausted cities in China can be calculated by the improved obstacle degree model and each obstacle factor is represented by a corresponding code. The results are shown in Table 13.

**Figure 3:**
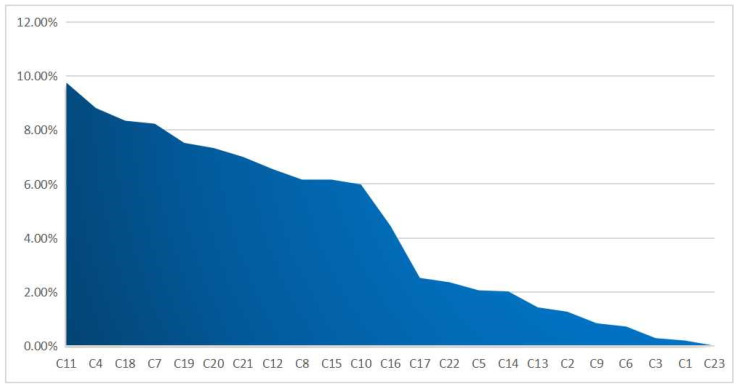
Diagram of the obstacle level of green development.

As can be seen from Table 13 and Figure 3, in terms of criteria level indicators, population and social welfare had the highest obstacle degree, reaching 39.53%, followed by 38.88% for economic development; the lowest barrier was for resources and the environment, reaching 21.59%. This shows that the cumulative factors related to the population’s social welfare and economic development hinder the green development of coal-resource-exhausted cities and the hindering effect on resources and the environment is smaller.

In terms of specific obstacle factors, among the 23 obstacle factors, 4 had an obstacle degree of more than 8% (C4, utilization rate of general industrial solid waste; C7, greening coverage rate of a built-up area; C11, proportion of science and technology in financial expenditure; C18, public buses/10,000 persons).

Seven obstacle factors were close to or more than 6% and less than 8% (C8, regional per capita GDP; C10, average annual salary of employees; C12, proportion of education in financial expenditure; C15, proportion of output value of the tertiary industry; C19, public library collection/100 persons; C20, the number of doctors/10,000 persons; C21, proportion of employed population in the primary industry) and one was 4%-5% (C16, population density).

Eleven obstacle factors were less than 3% (C1, emission of GRP industrial dust/CNY 100 million ; C2, emission of GRP industrial sulfur dioxide; C3, emission of GRP industrial wastewater/CNY 10,000 ; C5, centralized processing rate of sewage treatment plant; C6, harmless treatment rate of household trash; C9, growth rate of regional GDP; C13, proportion of output value of the primary industry; C14, proportion of output value of the secondary industry; C17, natural growth rate of population; C22, proportion of employed population in the secondary industry; C23, proportion of employed population in the tertiary industry).

Among C11, financial expenditure, the obstacle degree of the proportion of science and technology expenditure was the highest, at close to 10%. The factors with a high barrier degree and greater than 8% were the comprehensive utilization rate of C4, general industrial solid waste, the possession of public cars (electric vehicles) per 10,000 persons in C18 and the greening coverage rate in a built-up area, C7. In addition, there were other factors with high barriers, such as the collection of books in public libraries per 100 persons in C19, the number of doctors per 10,000 persons in C20, the proportion of employed population in primary industry in C21, the proportion of education expenditure in financial expenditure in C12, the per capita GDP of C8, the proportion of output value of the tertiary industry in C15, the average annual salary of employees in C10, etc.

In the criterion layer index of resources and the environment (21.59%), the obstacle degree of C1, emission of GRP industrial dust, was 0.20%; C2, emission of GRP industrial sulfur dioxide, was 1.27%; C3, emission of GRP industrial wastewater, was 0.29%; C5, centralized processing rate of sewage treatment plant, was 2.06%; and C6, harmless treatment rate of household trash, was 0.72%—all less than 3%. This has little impact on urban green development. In the criterion layer index of economic development (38.88%), the obstacle degree of C14, proportion of output value of the secondary industry, was 2.02%; C15, the proportion of output value of the tertiary industry, was 6.16, which means the impact on green development of the tertiary industry was larger than the secondary industry. Furthermore, in the criterion layer index of population and social welfare (39.53%), the obstacle degree of C16, population density, was up to 4.45%; C17, natural growth rate of population, was 2.52%, which shows that the population density has a great impact on urban development. This study has found that economic development and the population’s social welfare have greater impacts on urban sustainable development.

With regard to the characteristics of regions or specific obstacle factors between different groups of obstacle degrees, the characteristics of certain regional or specific obstacles exist between different groups or regions. For instance, due to geographical location and climatic conditions, the economy of coal-resource-exhausted cities in the western region of China is not as well developed as that of the eastern coastal groups, and social welfare (such as the number of books in public libraries per 100, the number of doctors per 10,000, etc.) and economic development (the proportion of science and technology in financial expenditure, the proportion of education expenditure, etc.) are the key factors affecting green development in these groups.

## 5. Discussion

### 5.1. Findings

First, the green development of coal-resource-exhausted cities in China has improved steadily and rapidly in the early stages. Through the calculation and analysis of the scores of the green development level, and the extreme value and mean value of coal-resource-exhausted cities in 2005, 2010 and 2015, it was shown that the level of green development showed a steady and upward trend from 2005 to 2015. Through the star-standard division and statistical analysis of the data, it was found that the level of green development from 2005 to 2010 was faster than that from 2010 to 2015, that is, the level increase in green development of coal-resource-exhausted cities in the first stage of the early stage was more than that in the second stage of the later stage; this trend was more obvious in coal-resource-exhausted cities with a lower number of stars.

Second, Zibo, Lanzhou and other cities with high levels of green development showed relatively stable green development. Through the calculation of the stability coefficient of data from the above three years, it was found that the stability index of the top five cities was 0.3, indicating that their ranking was relatively stable. The life cycle of resource-based cities, different effects of transformations on cities at different development stages and the evolution of the development stages of resource-based cities can directly reflect the changing process of urban development.

Third, the level of green development of most cities has improved; however, some cities have declined. Zibo was the city with the highest average green development and Laibin was the lowest. Wuhai made the fastest progress and Hegang made the slowest progress. By calculating the average level of green development in the three years, it was found that the three cities with the highest average level of green development were Zibo, Lanzhou and Xuzhou. The three cities with the lowest average level of green development were Laibin, Chaoyang and Chengde. During 11 years, the score ranking of green development in Wuhai increased by 23 places, and was 52% higher than that in 2005. Hegang, the city with the slowest progress, fell 20 places. In addition, it is worth noting that Baotou and Jingzhou regressed in green development and the level of green development in 2015 was lower than that in 2010.

Fourth, the transformation of 33 coal-resource-exhausted cities demonstrated certain regional characteristics. The green transformation and development of coal-resource-exhausted cities in China were mainly government-oriented. As the coal-resource-exhausted cities in the same province or region receive the same policy, plans for green transformation and development will be similar in the same region. Each coal-resource-exhausted city has different advantageous resources, which leads to different resource bases for green transformation, for example, Zaozhuang has built the Taierzhuang ancient town, which relies on canal culture, red cultural resources, etc., promoting city transformation through culture and tourism. Zibo, which is in the same province, also has rich cultural and tourism resources; however, the promotion of city transformation through culture and tourism is not appropriate here. Instead, high-value-added industries were developed, and now it has basically formed an industrial system dominated by four industries: building and new materials, medicine and fine chemicals, machinery manufacturing, and textiles and clothing. Hence, according to the regional characteristics, the results show that regional bias exists in resource-exhausted cities, and there are also biases in the same region.

Fifth, low financial expenditure on science and technology has become the primary reason for hindering green development. Based on the analysis of the obstacle degree, the obstacle degree of the proportion of science and technology expenditure in terms of total financial expenditure was close to 10%. There are also three factors with a high barrier degree that cannot be ignored: the comprehensive utilization rate of general industrial solid waste, the ownership of public buses (electric vehicles) per 10,000 persons and the greening coverage rate of a built-up area, which are reflected in the utilization of industrial waste, social welfare and urban greening, respectively. In addition, the factors with a high barrier degree also involve many aspects, such as medical treatment, agricultural population, education, regional GDP, tertiary industry, wages and salaries, etc. Moreover, ecological remediation is an important task in the economic transformation of coal-resource-exhausted cities. In this research, we studied barriers to green development in coal-resource-exhausted cities. It was found that factors related to the population’s social welfare and economic development have a great impact on the green development of resource-exhausted cities, and the low proportion of financial investment into science and technology is the main cause. The research shows that continuously increasing financial investment in the areas of scientific and technological innovation and establishing a diversified mechanism can promote the transformation of new and old kinetic energy, fundamentally improve the ecological environment in coal-resource-exhausted cities and achieve green development.

### 5.2. Theoretical Contributions

Compared with previous research, our study contributes to the green sustainable development of resource-exhausted cities in the following ways:

First, we explore a new research perspective (i.e., evaluation index system and obstacle degree analysis based on the green development theory) to focus on resource-exhausted cities that are facing resource depletion, environmental problems, the recession of industry and imbalanced unemployment, etc. [80]. Based on the principal component analysis of resource-exhausted cities, this study proposed and constructed an evaluation index system for the green sustainable development of coal-resource-exhausted cities. More concretely, two of the most important processes in constructing the evaluation index system of green development for coal-resource-exhausted cities are the selection and weighting of indicators. Based on the analysis of characteristics of coal-resource-exhausted cities and basic principles of selecting indicators of green development, an evaluation index system of green development for coal-resource-exhausted cities was built.

Second, this study has filled the research gap in terms of the theory of green sustainable development in resource-exhausted cities and improved it further by a series of empirical studies and analyses, providing relative research results. It has been demonstrated that green development has a positive impact on improving social and ecological environmental problems [62,81,82]. Compared with previous research, the innovation of this study is that, following on from the sustainable development needs, we establish that the five development concepts of “innovation, coordination, green, openness and sharing” have important significance for urban transformation. Guided by the five development concepts, we are now able to ensure the green development of coal-resource-exhausted cities with innovation as the driving force, coordination as the content, green as the goal, open as the trend and sharing as the requirements, and provide a decision-making basis for economic transformation and development with a view to exploring the green development path of coal-resource-exhausted cities on the basis of social and economic development and the ecological environment.

Third, this study adopted both the qualitative and quantitative analysis method. The qualitative analysis method was used for the selection of indicators and selected indicators scientifically and reasonably. The quantitative analysis method was adopted for index weighting to avoid artificial bias and subjective factors affecting the objectivity of index weighting. Therefore, with the goal of green and sustainable development, this study builds a three-level index system by using development analysis tools and a principal component extraction method.

Finally, based on the results of our empirical analysis, this study discusses the life cycle of resource-based cities [83,84] and the different effects of transformation on cities at different development stages; it also makes recommendations accordingly to improve the green development of cities [85]. We argue that the obstacle degree can act as a basis for the judgement and evaluation of resource-exhausted cities. Furthermore, through the star-standard division and statistical analysis of the data for the green development of resource-exhausted cities, it can be explicated that the methods and analyses concerning the green sustainable development of cities have an active response to urban development and the improvement of the ecological environment [86].

### 5.3. Recommendation

According to the actual evaluation situation of the green development of coal-resource-exhausted cities and the discussion regarding the life cycle of resource-based cities combined with the conclusions regarding the obstacle degree analysis, the following recommendations are drawn:

First, science and technology are core components of green development. The biggest obstacle for coal-resource-exhausted cities in achieving green development is the lack of scientific and technological financial expenditure [87]. On one hand, increasing financial investment, especially science and technology expenditure, can promote the transformation of old and new kinetic energy and provide industrial upgrades [88]. On the other hand, scientific and technological means can be used to improve the scientific and technological content of resource-based industry, expand the industrial chain and accelerate the transformation and upgrading of resource-based industry, which are all key for green development. Furthermore, encouraging and guiding the development of tertiary industry with scientific and technological innovation is also important. The service industry is the core of the industrial structure adjustment of resource-based cities and benefits the development of tertiary industry [89]. It can alleviate employment pressure and absorbs a large number of workers who leave, retire, are laid-off or transfer from resource-based industries; furthermore, it is also easier to promote scientific and technological innovation and realize the virtuous cycle of urban green development in the development of tertiary industry [89,90].

Second, prosperity is associated with improving welfare. Achieving green development not only “enriches the country” but also “enriches the people”. On the basis of green production, improving welfare is another important part of green development. The measures include establishing and improving public transport systems and the use of new energy. Moreover, the government should also increase investment in public healthcare and education, focusing on increasing the number of community doctors and reducing the medical pressures on large hospitals.

The third conclusion concerns environmental protection and ecological restoration. The ecological environment of coal-resource-exhausted cities is generally poor [91]. Therefore, improving the ecological environment has become the core measure to ensure green development, which includes improving the recycling rate of industrial solid waste and reducing ecological pollution. Since industrial solid waste generally originates from enterprises, it is more conducive to the development of enterprises if they introduce market-oriented means to solve the problem of solid waste utilization [92]. This includes actively introducing or establishing professional solid-waste treatment companies to treat industrial solid waste [93,94]. Due to long-term coal mining, the ecological environment in coal-resource-exhausted cities is seriously damaged, and the dust pollution and collapse in mining areas are prominent; therefore, constructing mining parks and afforestation, and restoring the urban ecology are key to building ecological restoration in these cities.

### 5.4. Limitations and Future Directions

Based on the characteristics of typical resource-exhausted cities, this study tried to reveal the impacts of different regions, as well as reflect regional variations, on urban transformation. We further analyzed the obstacle factors and obstacle degree of green development on resource-exhausted cities by empirical analysis. Furthermore, the stage characteristics of the evolution and life cycle of coal-resource-exhausted cities are also discussed in this study in order to explore the ways of green sustainable development. There are several limitations to be noted that also provide directions for future studies.

First, affected by the limitations of data acquisition, this study only evaluated the green development level of 33 coal-resource-exhausted cities, and in a future study, we will continue to track the latest data to continuously evaluate the green development level of more cities with significant regional characteristics, as well as the impact of the barriers in different regions on green sustainable development.

Second, principal component analysis was mainly adopted in this study, which can ensure that the weight of each index completely depends on the objective information during the calculation process, avoiding the deviation caused by subjective factors. Meanwhile, on the premise of avoiding the loss of information, the high-dimensional index vector was reduced, simplifying the multivariate panel data and reducing the workload. However, this method also has some problems. The principal component analysis is carried out from the correlation matrix of the sample, that is, the original data should be standardized, but the standardization eliminates the variation degree of each variable while eliminating the dimension or order of magnitude. Moreover, extracting the variance contribution rate of each principal component as the weight to calculate a comprehensive score will have certain subjective components. Hence, the entropy method, integrated comprehensive evaluation, principal component clustering method, etc., may be used to improve this study in the future.

Third, during the construction of the evaluation index system of green development for coal-resource-exhausted cities, indicators concerning environmental pollution will inevitably be involved, which are also the core factors affecting the green transformation and development. In the process of the field investigation, the data were from 33 cities over a 10-year period, which may mean there are missing or fuzzy data. This makes it more difficult to obtain objective first-hand data. In a future study, according to the development and improvement of government, enterprise and public awareness of green development, each index layer of the evaluation index system of green development could be appropriately adjusted for a more comprehensive and systematic analysis.

In addition, according to the degree of green development of cities, the development stages of cities can be classified into several types; however, this study is mainly focused on resource-exhausted cities, ignoring both growing and mature resource-based cities. The core of urban construction is always how to achieve the green sustainable development. Thus, a future study will further expand the green development levels of mature resource-based cities and growing resource-based cities.

## 6. Conclusions

On the basis of analyzing the characteristics of coal-resource-exhausted cities, this study builds an evaluation index system of green development that can provide a reference for the green transformation and sustainable development of resource-exhausted cities. Based on theoretical reviews, empirical analysis and discussion, the following conclusion can be drawn from this study:

Urban construction should move towards a composite system of “production, ecology, life”. Improving the ecological environment, promoting green low-carbon industries and realizing circular development will become the pursuit of green urban development. Building a green city is a systematic project, gradually realizing the prosperity of green production, the benefits of sharing and symbiosis with green living, reaching a consensus and reconciliation between humans and nature [95]. There is no end to the construction of green cities; people should create a better life with intensive, low-carbon and high-quality development in the construction process in the long term. Moreover, in order to improve the declining situation and promote urban sustainability, resource-exhausted cities must follow the sustainable development rules of the economy, society and the ecological environment so as to save the city and escape “the valley of death”.

## Figures and Tables

**Table 1:** Transformation cases of resource-exhausted cities.

Country	Cases	Resources	Transformation Modes
Germany	Ruhr Industrial Base	Resource-based production (coal and steel)	New economic zone (computer and information technology industry, etc.)
France	Lorraine	Chemical industry (iron ore and coal mine)	Industrial transformation
The United States	Pittsburgh	Steel-resource-based city	Light and service industry
The United States	Houston	Oil-resource-based city	Comprehensive metropolis (aerospace center)
The United States	Los Angeles	Oil-resource-based city	Agriculture, aircraft manufacturing, ordnance industries and clusters
Japan	Kyushu	Coal-resource-based city	High-tech industrial zones
Spain	Ibérian	Mining area	New geological mining
Italy	Italy	Mining industry	Geology and urban sustainability
Greece	Peloponnese Peninsula	Coal mine Mining	Mining reclamation, modern agriculture

**Table 2:** Policy orientations of transformation cases of resource-exhausted cities.

Country	Type	Characteristics
The United States	Market-oriented	The government rarely takes specific transformational control, whether the city prospers or declines is more determined by market forces and enterprises’ development goals.
The EU	Government-oriented	The government establishes special committees to formulate detailed objectives, plans and policies, adjust the industrial structure, promote regional industrial progress and economic development, and realize the take-off of the regional economy through the full cooperation of government and society.
Japan	Industrial policy	A kind of industrial aid under the guidance of industrial policy. The government formulates and modifies industrial policies, sets goals and measures according to the changes of domestic and foreign markets and the situation in coal areas.
South America (Venezuela, etc.)	Laissez-faire	The government has hardly taken any transformation measures, so the development of resource-based cities has to stagnate.
China	Government-oriented	Once identified as a resource-exhausted city by the State Council, the government will provide financial transfer support.

**Table 3:** Comparison of relative research methods and conclusions.

Reference	Case Study	Methodology	Data Source	Research Content
Zeng and Duan (2018) [64]	Study on performance evaluation of green transformation of coal-resource-exhausted cities	Cluster analysis	Data analysis	The research was focused on differences in green transformation performance among different cities.
Liu et al. (2020) [65]	Evaluation on sustainable development of coal-resource-exhausted cities	SBM DEA model/Malmquist index model	Respondent	The evaluation was based on the transformation efficiency of sustainable development.
Tao et al. (2022) [66]	Performance measurement and obstacle factor analysis of the transformation of coal-resource-exhausted cities	Obstacle factor analysis	Statistics	A total of 27 indicators were selected from the three dimensions of economic development, people’s wellbeing and ecological environment to build a measurement system, and Jiaozuo City was taken as an example to measure the performance of transformation and development in 2008–2018.
Dwivedi and Sharma (2022) [67]	Shannon entropy and COCOSO techniques to analyze the performance of sustainable development goals: The case of the Indian Union Territories	Entropy-based method/ COCOSO method	Official statistics	This study examined key targets for Indian Union Territories using the SDG India Index 3.0 and the proposed technology, as well as being based on demonstration of the assessed union territories with their achieving rank. Shannon entropy and COCOSO techniques were used in the MCDM model for evaluating the target.
Yu (2022) [68]	Evaluation of the green transformation model for coal-resource-exhausted cities	Analytic hierarchy process (AHP)	Data analysis	This study used the analytic hierarchy process to evaluate the effect on the green development of four types of green transformation models, and put forward the strategies of the green transformation model in coal-resource-exhausted cities.
Dewa et al. (2022) [69]	Shannon entropy-based urban spatial fragmentation to ensure the sustainable development of the urban coastal city: A case study of Semarang, Indonesia	Entropy-based methods	USGS website	This study examined the extent to which the Shannon entropy index (H) could be used to ensure urban growth sustainability by measuring the spatial dispersion pattern of a built-up area. The Shannon entropy index was calculated based on the proximities to the city center (HCC) and the main road (HMR), which divided the study area into 17 zones.
Saiu et al. (2022) [70]	Making sustainability development goals (SDGs) operational at a suburban level	SDG–NSA cross-analysis	Comparative analysis	This study aimed to contribute to fill this gap by examining the usefulness of neighborhood sustainability assessment (NSA) tools for operationalizing the 17 SDGs.
Cunha-Zeri et al. (2022) [71]	A sustainability assessment using the entropy weight method in Brazil	Entropy weight method	Official sources	This study conducted an assessment of nitrogen sustainability in Brazil from 2000 to 2018, applying the entropy weight method (EWM) to a set of nitrogen-related indicators within four subsystems: environmental, economic, social and institutional.
This paper	Evaluation index system and obstacle degree analysis on green sustainable development	Principal component analysis/extremum method	Field survey/ models	This study used the star-level standards on the scores of green development from 2005 to 2015. Through statistical analysis, the level and obstacles of urban green development were obtained.

**Table 4:** Distribution of coal-resource-exhausted cities in China.

Region	Coal-Resource-Exhausted Cities
Northeast	Fuxin, Fushun, Liaoyuan, Hegang, Shuangyashan, Qitaihe, Changchun, Jilin, Tonghua, Chaoyang
East	Zaozhuang, Tai’an, Zibo, Xuzhou, Pingxiang, Huaibei
North	Zhangjiakou, Chengde, Linfen, Wuhai, Baotou
Central	Jiaozuo, Jingzhou, Hengyang, Chenzhou, Loudi
South	Shaoguan
Southwest	Laibin, Chongqing, Guang’an
Northwest	Shizuishan, Tongchuan, Lanzhou

Source: The National Sustainable Development Plan for Resource-based Cities (2013–2020) (GF [2013] No. 45) issued by the State Council in 2013 and the Statistical Yearbook of Cities in 2020.

**Table 5:** Evaluation index system of green development for coal-resource-exhausted cities.

Target Layer	Rule Layer	Index Layer	Unit	Index Properties
**Evaluation index system of green development for coal-resource-exhausted cities**	Resources and the environment	Emission of GRP industrial dust /CNY 100 million	Ton/CNY 100 million	Negative
		Emission of GRP industrial sulfur dioxide /CNY 100 million	Ton/CNY 100 million	Negative
		Emission of GRP industrial wastewater /CNY 10,000	Ton/CNY 100 million	Negative
		Utilization rate of general industrial solid waste	%	Positive
		Centralized processing rate of sewage treatment plant	%	Positive
		Harmless treatment rate of household trash	%	Positive
		Greening coverage rate of a built-up area	%	Positive
	Economic development	Regional per capita GDP	CNY	Positive
		Growth rate of regional GDP	%	Positive
		Average annual salary of employees	CNY	Positive
		Proportion of science and technology in financial expenditure	‰	Positive
		Proportion of education in financial expenditure	‰	Positive
		Proportion of output value of primary industry	%	Positive
		Proportion of output value of secondary industry	%	Positive
		Proportion of output value of tertiary industry	%	Positive
	Population, social welfare	Population density	Person/km^2^	Positive
		Natural growth rate of population	‰	Positive
		Public buses (electric vehicles) /10,000 persons	Vehicle	Positive
		Public library collection/100 persons	Volume	Positive
		the number of doctors/10,000 persons	Person	Positive
		Proportion of employed population in the primary industry	%	Positive
		Proportion of employed population in the secondary industry	%	Positive
		Proportion of employed population in the tertiary industry	%	Positive

**Table 6:** KMO and Bartlett’s test of sphericity.

KMO Test and Bartlett’s Test of Sphericity		Test Value
**Kaiser–Meyer–Olkin measure of sampling adequacy**		0.589
**Bartlett’s test of sphericity**	Chi-square test	10,479
	Df	253
	Sig.	0.000

**Table 7:** Principal component load.

Index Variable	PC1	PC2	PC3	PC4	PC5	PC6
Emission of GRP industrial dust /CNY 100 million	0.0891	0.123	0.0661	−0.0753	0.248	−0.244
Emission of GRP industrial sulfur dioxide/CNY 100 million	0.195	0.187	0.171	0.0428	0.487	−0.107
Emission of GRP industrial wastewater/CNY 10,000	0.199	0.119	−0.0446	−0.120	0.534	−0.147
Utilization rate of general industrial solid waste	0.0561	−0.102	0.172	0.405	0.115	0.267
Centralized processing rate of sewage treatment plant	0.272	0.0354	0.198	0.142	−0.153	−0.246
Harmless treatment rate of household trash	0.140	−0.0880	0.152	0.0542	−0.270	−0.447
Greening coverage rate of a built-up area	0.0372	−0.0175	0.00727	−0.0868	0.113	−0.124
Regional per capita GDP	0.371	0.0883	−0.0602	0.0151	−0.210	−0.0363
Growth rate of regional GDP	−0.0906	−0.283	−0.252	−0.239	0.158	0.112
Average annual salary of employees	0.310	0.179	0.231	0.113	−0.218	−0.147
Proportion of science and technology in financial expenditure	0.286	−0.0518	0.173	0.130	0.0221	0.138
Proportion of education in financial expenditure	−0.0925	−0.0440	0.312	0.219	0.0664	0.315
Proportion of output value of primary industry	−0.342	0.217	0.0743	0.269	−0.0293	−0.184
Proportion of output value of secondary industry	0.212	−0.439	−0.141	−0.151	0.0171	−0.156
Proportion of output value of tertiary industry	0.142	0.343	0.104	−0.135	0.0136	0.463
Population density	0.0885	−0.351	0.341	0.146	0.235	0.0562
Natural growth rate of population	0.00367	−0.226	0.296	−0.120	−0.220	0.201
Public buses (electric vehicles) /10,000 persons	0.179	0.247	−0.0507	−0.0863	0.115	0.0560
Public library collection/100 persons	0.247	0.108	−0.145	−0.163	−0.162	0.219
the number of doctors/10,000 persons	0.247	0.218	−0.331	0.0842	−0.135	0.149
Proportion of employed population in the primary industry	−0.178	0.168	−0.268	0.465	0.0289	−0.0795
Proportion of employed population in the secondary industry	0.283	−0.284	−0.203	0.169	0.0549	0.0937
Proportion of employed population In the tertiary industry	−0.168	0.175	0.372	−0.463	−0.0729	−0.0427

Abbreviation of principal component in the table: PC.

**Table 8:** Principal component load.

Index Variable	PC7	PC8	PC9	PC10	PC11	PC12
Emission of GRP industrial dust /CNY 100 million	−0.333	0.0121	0.493	0.537	0.299	−0.187
Emission of GRP industrial sulfur dioxide/CNY 100 million	0.132	−0.0463	0.000340	−0.237	−0.0222	0.0940
Emission of GRP industrial wastewater/CNY 10,000	0.0545	−0.124	0.0833	−0.0654	−0.267	0.165
Utilization rate of general industrial solid waste	−0.201	0.360	0.272	−0.265	0.0847	0.297
Centralized processing rate of sewage treatment plant	0.211	0.0331	−0.162	−0.0513	−0.0127	−0.0937
Harmless treatment rate of household trash	−0.112	0.188	−0.00261	−0.276	0.448	0.0905
Greening coverage rate of a built-up area	0.125	0.787	−0.296	0.339	−0.258	0.0723
Regional per capita GDP	0.0845	0.0115	0.105	0.0670	−0.0348	−0.126
Growth rate of regional GDP	0.222	0.229	0.0177	−0.00868	0.423	−0.110
Average annual salary of employees	0.00459	−0.0394	0.0217	0.0701	−0.187	0.0744
Proportion of science and technology in financial expenditure	0.239	−0.0217	−0.0692	0.217	0.105	−0.466
Proportion of education in financial expenditure	0.492	−0.103	0.0771	0.0965	0.137	−0.0873
Proportion of output value of primary industry	−0.0258	0.0188	−0.00613	0.0327	0.0288	−0.0398
Proportion of output value of secondary industry	0.233	−0.118	0.119	0.00727	−0.0623	0.158
Proportion of output value of tertiary industry	−0.298	0.143	−0.162	−0.0522	0.0513	−0.172
Population density	−0.0767	0.00965	−0.0174	−0.00262	0.0707	0.0453
Natural growth rate of population	−0.106	−0.136	0.0425	0.444	−0.0965	0.488
Public buses (electric vehicles) /10,000 persons	0.0495	−0.145	−0.442	0.135	0.540	0.400
Public library collection/100 persons	0.146	0.215	0.470	−0.182	0.0278	0.0590
the number of doctors/10,000 persons	0.137	−0.0199	0.0924	0.0902	0.00286	0.234
Proportion of employed population in the primary industry	0.238	0.0422	0.121	0.213	0.0534	0.157
Proportion of employed population in the secondary industry	−0.329	−0.0777	−0.204	−0.0109	−0.0489	−0.141
Proportion of employed population In the tertiary industry	0.176	0.0504	0.126	−0.124	0.0147	0.0405

Abbreviation of principal component in the table: PC.

**Table 9:** Evaluation index system of green development of coal-resource-exhausted cities after empowerment.

	Rule Layer	Index Layer	Index Weight (%)
**Evaluation index system of green development for coal-resource-exhausted cities**	Resources and the environment (0.354)	Emission of GRP industrial dust /CNY 100 million	0.062
		Emission of GRP industrial sulfur dioxide /CNY 100 million	0.079
		Emission of GRP industrial wastewater /CNY 10,000	0.042
		Utilization rate of general industrial solid waste	0.090
		Centralized processing rate of sewage treatment plant	0.034
		Harmless treatment rate of household trash	0.006
		Greening coverage rate of a built-up area	0.041
	Economic development (0.33)	Regional per capita GDP	0.039
		Growth rate of regional GDP	0.013
		Average annual salary of employees	0.052
		Proportion of science and technology in financial expenditure	0.067
		Proportion of education in financial expenditure	0.083
		Proportion of output value of primary industry	0.011
		Proportion of output value of secondary industry	0.025
		Proportion of output value of tertiary industry	0.049
	Population, social welfare (0.308)	Population density	0.038
		Natural growth rate of population	0.021
		Public buses (electric vehicles) /10,000 persons	0.066
		Public library collection/100 persons	0.058
		the number of doctors/10,000 persons	0.054
		Proportion of employed population in the primary industry	0.043
		Proportion of employed population in the secondary industry	0.028
		Proportion of employed population in the tertiary industry	0.000

**Table 10:** Evaluation scores of green development of coal-resource-exhausted cities from 2005 to 2015.

No.	City	2005	2006	2007	2008	2009	2010	2011	2012	2013	2014	2015
1	Zhangjiakou	0.340	0.361	0.385	0.395	0.447	0.439	0.442	0.429	0.451	0.450	0.486
2	Chengde	0.358	0.351	0.419	0.465	0.429	0.420	0.462	0.423	0.434	0.433	0.452
3	Linfen	0.364	0.383	0.444	0.446	0.458	0.424	0.440	0.450	0.453	0.445	0.471
4	Baotou	0.396	0.465	0.523	0.514	0.544	0.565	0.579	0.551	0.559	0.582	0.554
5	Wuhai	0.354	0.409	0.420	0.428	0.482	0.499	0.496	0.499	0.556	0.532	0.570
6	Fushun	0.390	0.415	0.424	0.430	0.431	0.440	0.455	0.458	0.450	0.463	0.479
7	Fuxin	0.372	0.365	0.375	0.416	0.400	0.420	0.414	0.436	0.424	0.428	0.448
8	Chaoyang	0.347	0.336	0.379	0.411	0.356	0.385	0.418	0.419	0.420	0.438	0.439
9	Changchun	0.443	0.460	0.493	0.484	0.512	0.555	0.525	0.561	0.576	0.577	0.568
10	Jilin	0.376	0.406	0.441	0.461	0.480	0.466	0.481	0.483	0.506	0.518	0.493
11	Liaoyuan	0.385	0.457	0.427	0.467	0.475	0.486	0.450	0.482	0.500	0.490	0.494
12	Tonghua	0.393	0.389	0.454	0.410	0.456	0.456	0.468	0.499	0.542	0.513	0.524
13	Hegang	0.430	0.405	0.462	0.466	0.454	0.464	0.470	0.463	0.480	0.467	0.467
14	Shuangyashan	0.408	0.412	0.459	0.467	0.437	0.425	0.423	0.349	0.439	0.455	0.532
15	Qitaihe	0.405	0.414	0.448	0.442	0.414	0.427	0.436	0.463	0.458	0.461	0.444
16	Xuzhou	0.464	0.477	0.508	0.514	0.518	0.526	0.525	0.558	0.588	0.597	0.601
17	Huaibei	0.443	0.443	0.483	0.502	0.497	0.527	0.536	0.538	0.530	0.543	0.532
18	Pingxiang	0.389	0.405	0.435	0.438	0.438	0.448	0.465	0.478	0.511	0.538	0.534
19	Zibo	0.478	0.490	0.560	0.567	0.552	0.574	0.600	0.605	0.625	0.617	0.629
20	Zaozhuang	0.432	0.451	0.483	0.502	0.507	0.522	0.523	0.521	0.528	0.546	0.550
21	Tai’an	0.444	0.480	0.510	0.560	0.513	0.519	0.530	0.536	0.559	0.560	0.577
22	Jiaozuo	0.407	0.414	0.457	0.473	0.498	0.512	0.509	0.506	0.523	0.532	0.529
23	Jingzhou	0.407	0.414	0.441	0.462	0.461	0.502	0.462	0.462	0.427	0.452	0.476
24	Hengyang	0.378	0.387	0.418	0.440	0.435	0.453	0.464	0.472	0.477	0.498	0.506
25	Chenzhou	0.399	0.385	0.413	0.433	0.456	0.464	0.465	0.473	0.482	0.510	0.528
26	Loudi	0.407	0.386	0.420	0.391	0.433	0.444	0.442	0.480	0.482	0.476	0.491
27	Shaoguan	0.365	0.404	0.432	0.459	0.462	0.480	0.497	0.496	0.497	0.514	0.530
28	Laibin	0.297	0.290	0.289	0.358	0.361	0.402	0.431	0.422	0.462	0.473	0.449
29	Chongqing	0.422	0.402	0.448	0.457	0.444	0.504	0.467	0.466	0.513	0.519	0.520
30	Guang’an	0.332	0.359	0.401	0.414	0.427	0.455	0.454	0.468	0.461	0.447	0.475
31	Tongchuan	0.386	0.413	0.414	0.424	0.446	0.440	0.480	0.500	0.513	0.521	0.529
32	Lanzhou	0.481	0.517	0.559	0.556	0.549	0.561	0.574	0.595	0.547	0.562	0.587
33	Shizuishan	0.311	0.320	0.362	0.419	0.456	0.434	0.439	0.456	0.474	0.497	0.516

**Table 11:** Score and rank of green development of coal-resource-exhausted cities in 2005, 2010 and 2015.

No.	City	Rank 2005	Rank 2010	Rank 2015
1	Zhangjiakou	30	25	23
2	Chengde	27	31	29
3	Linfen	26	29	27
4	Baotou	16	2	7
5	Wuhai	28	12	5
6	Fushun	18	24	24
7	Fuxin	24	30	31
8	Chaoyang	29	33	33
9	Changchun	5	4	6
10	Jilin	23	15	21
11	Liaoyuan	21	13	20
12	Tonghua	17	18	16
13	Hegang	8	17	28
14	Shuangyashan	10	28	10
15	Qitaihe	14	27	32
16	Xuzhou	3	6	2
17	Huaibei	6	5	11
18	Pingxiang	19	21	9
19	Zibo	2	1	1
20	Zaozhuang	7	7	8
21	Tai’an	4	8	4
22	Jiaozuo	13	9	14
23	Jingzhou	12	11	25
24	Hengyang	22	20	19
25	Chenzhou	15	16	15
26	Loudi	11	22	22
27	Shaoguan	25	14	12
28	Laibin	33	32	30
29	Chongqing	9	10	17
30	Guang’an	31	19	26
31	Tongchuan	20	23	13
32	Lanzhou	1	3	3
33	Shizuishan	32	26	18

**Table 12:** Division of green development level of coal-resource-exhausted cities.

Level of Green Development	One Star	Two Stars	Three Stars	Four Stars
Score of green development	[0, 0.424]	[0.424, 0.461]	[0.461, 0.508]	[0.508, 1]
Representation state	Poor	Commonly	Good	Excellent

**Table 13:** Obstacle factors and degree of green development of coal-resource-exhausted cities.

Criterion Layer Indexand Obstacle Degree	Obstacle Factors	Obstacle Code	Obstacle Degree
Resources and the environment (21.59%)	Emission of GRP industrial dust /CNY 100 million	C1	0.20%
	Emission of GRP industrial sulfur dioxide /CNY 100 million	C2	1.27%
	Emission of GRP industrial wastewater /CNY 10,000	C3	0.29%
	Utilization rate of general industrial solid waste	C4	8.81%
	Centralized processing rate of sewage treatment plant	C5	2.06%
	Harmless treatment rate of household trash	C6	0.72%
	Greening coverage rate of a built-up area	C7	8.23%
	Regional per capita GDP	C8	6.16%
Economic development (38.88%)	Growth rate of regional GDP	C9	0.84%
	Average annual salary of employees	C10	5.98%
	Proportion of science and technology in financial expenditure	C11	9.75%
	Proportion of education in financial expenditure	C12	6.55%
	Proportion of output value of the primary industry	C13	1.43%
	Proportion of output value of the secondary industry	C14	2.02%
	Proportion of output value of the tertiary industry	C15	6.16%
Population’s social welfare (39.53%)	Population density	C16	4.45%
	Natural growth rate of population	C17	2.52%
	Public buses (electric vehicles) /10,000 persons	C18	8.34%
	Public library collection/100 persons	C19	7.52%
	The number of doctors/10,000 persons	C20	7.33%
	Proportion of employed population in the primary industry	C21	7.00%
	Proportion of employed population in the secondary industry	C22	2.36%
	Proportion of employed population in the tertiary industry	C23	0.01%

GRP refers to the gross regional product.

## Data Availability

Not applicable.

## References

[1] Zhu Y., Zhang R., Cui J. (2022). Spatial Differentiation and Influencing Factors in the Ecological Well-Being Performance of Urban Agglomerations in the Middle Reaches of the Yangtze River: A Hierarchical Perspective. Int. J. Environ. Res. Public Health.

[2] Omri A. (2020). Technological innovation and sustainable development: Does the stage of development matter?. Environ. Impact Assess..

[3] Zhai X.Q., An Y.F. (2021). The relationship between technological innovation and green transformation efficiency in China: An empirical analysis using spatial panel data. Technol. Soc..

[4] Majerník M., Naščáková J., Malindžáková M., Drábik P., Bednárová L. (2021). Areas of sustainability: Environment, economy, and society. Sustainable Resource Management.

[5] Ogunmakinde O.E., Egbelakin T., Sher W. (2022). Contributions of the circular economy to the UN sustainable development goals through sustainable construction. Resour. Conserv. Recycl..

[6] Awosusi A.A., Xulu N.G., Ahmadi M., Rjoub H., Altuntaş M., Uhunamure S.E., Akadiri S.S., Kirikkaleli D. (2022). The Sustainable environment in Uruguay: The roles of financial development, natural resources, and trade globalization. Front. Environ. Sci..

[7] Liu S.M., Gao L.F., Hu X.H., Shi J., Mohsin M. (2022). Does industrial eco-innovative development and economic growth affect environmental sustainability? New evidence from BRICS countries. Front. Environ. Sci..

[8] Nogueira C. (2019). Contradictions in the concept of sustainable development: An analysis in social, economic, and political contexts. Environ. Dev..

[9] Jacobson M.Z., Krauland A.K.V., Coughlin S.J., Palmer F.C., Smith M.M. (2022). Zero air pollution and zero carbon from all energy at low cost and without blackouts in variable weather throughout the U.S. with 100% wind-water-solar and storage. Renew. Energy.

[10] Strokal V., Kuiper E.J., Bak M.P., Vriend P., Wang M.R., Wijnen J.V., Strokal M. (2022). Future microplastics in the Black Sea: River exports and reduction options for zero pollution. Mar. Pollut. Bull..

[11] Capasso M., Hansen T., Heiberg J., Klitkou A., Steen M. (2019). Green growth–A synthesis of scientific findings. Technol. Forecast. Soc. Chang..

[12] Bonsu N.O. (2020). Towards a circular and low-carbon economy: Insights from the transitioning to electric vehicles and net zero economy. J. Clean. Prod..

[13] Vargas-Hernandez J.G. (2020). Strategic transformational transition of green economy, green growth, and sustainable development: An institutional approach. Int. J. Green Comput..

[14] Sun Y., Ding W., Yang Z., Yang G., Du J. (2020). Measuring China’s regional inclusive green growth. Sci. Total Environ..

[15] Zhou G., Zhu J., Luo S. (2022). The impact of fintech innovation on green growth in China: Mediating effect of green finance. Ecol. Econ..

[16] Jamei E., Jamei Y., Seyedmahmoudian M., Horan B., Mekhilef S., Stojcevski A. (2022). Investigating the impacts of COVID-19 lockdown on air quality, surface Urban Heat Island, air temperature and lighting energy consumption in City of Melbourne. Energy Strategy Rev..

[17] Wang Y.J., Chen H., Long R.Y., Sun Q.Q., Jiang S.Y., Liu B. (2022). Has the Sustainable Development Planning Policy Promoted the Green Transformation in China’s Resource-based Cities?. Resour. Conserv. Recycl..

[18] Zhou Q., Zhu M.K., Qiao Y., Zhang X., Chen J. (2021). Achieving resilience through smart cities? Evidence from China. Habitat Int..

[19] Guo J., Chen Y.Y., Hao D.P., Zhang L.J. (2022). A multi-criteria decision-making approach to help resource-exhausted areas choose suitable transformation templates—The example of Wansheng in Chongqing, China. Ain Shams Eng. J..

[20] Mardones C., Ricardo D.R. (2019). Correction of Chilean GDP for natural capital depreciation and environmental degradation caused by copper mining. Resour. Policy.

[21] Venter Z.S., Figari H., Krange O., Gundersen V. (2023). Environmental justice in a very green city: Spatial inequality in exposure to urban nature, air pollution and heat in Oslo, Norway. Sci. Total Environ..

[22] Long R.Y., Li H.F., Wu M.F., Li W.B. (2021). Dynamic evaluation of the green development level of China’s coal-resource-based cities using the TOPSIS method. Resour. Policy.

[23] Wang D.L., Huang Z.Y., Wang Y.D., Mao J.Q. (2021). Ecological security of mineral resource-based cities in China: Multidimensional measurements, spatiotemporal evolution, and comparisons of classifications. Ecol. Indic..

[24] Sun X., Zhu B.K., Zhang S., Zeng H., Li K., Wang B., Dong Z., Zhou C. (2022). New indices system for quantifying the nexus between economic-social development, natural resources consumption, and environmental pollution in China during 1978–2018. Sci. Total Environ..

[25] Yang Y., Guo H., Chen L., Liu X., Gu M., Ke X. (2019). Regional analysis of the green development level differences in Chinese mineral resource-based cities. Resour. Policy.

[26] Chapman R., Plummer P., Tonts M. (2015). The resource boom and socio-economic well-being in Australian resource towns: A temporal and spatial analysis. Urban Geogr..

[27] Chen W., Shen Y., Wang Y.A. (2018). Evaluation of economic transformation and upgrading of resource-based cities in Shaanxi province based on an improved TOPSIS method. Sustain. Cities Soc..

[28] Falck O., Koenen J., Lohse T. (2019). Evaluating a place-based innovation policy: Evidence from the innovative regional growth cores program in East Germany. Reg. Sci. Urban Econ..

[29] Lu H.Y., Liu M., Song W.J. (2022). Place-based policies, government intervention, and regional innovation: Evidence from China’s Resource-Exhausted city program. Resour. Policy.

[30] Fang X.C., Chen H. (2016). Measurement on the efficiency of economic transformation for the resource declining cities. Urban Probl..

[31] Shao J., Zhou J.Q. (2011). Study on the influences of industry transformation on the sustainable development of resource-exhausted city space. Procedia Eng..

[32] Guo Q.B., Meng X.R., Li Y.M., Lv X., Liu C. (2020). A prediction model for the surface residual subsidence in an abandoned goaf for sustainable development of resource-exhausted cities. J. Clean. Prod..

[33] Yang B., Zhan X.Y., Tian Y.H. (2021). Evaluation on the effect of the transformation policy of resource-exhausted cities—An empirical analysis based on the difference-in-difference model. Energy Rep..

[34] Namazi M., Mohammadi E. (2018). Natural resource dependence and economic growth: A TOPSIS/DEA analysis of innovation efficiency. Resour. Policy.

[35] Basu T., Das A. (2021). Systematic review of how eco-environmental transformation due to urbanization can be investigated in the sustainable development of Indian cities. Environ. Chall..

[36] Lotz-Sisitka H., Ali M.B., Mphepo G., Chaves M., Macintyre T., Pesanayi T., Wals A., Mukute M., Kronlid D., Tran D.T. (2016). Co-designing research on transgressive learning in times of climate change. Curr. Opin. Environ. Sustain..

[37] El-gafy I., Grigg N., Waskom R. (2017). Water-food-energy: Nexus and non-nexus approaches for optimal cropping pattern. Water Resour. Manag..

[38] Pauliuk S., Koslowski M., Madhu K., Schulte S., Kilchert S. (2022). Co-design of digital transformation and sustainable development strategies—What socio-metabolic and industrial ecology research can contribute. J. Clean. Prod..

[39] Sebri M. (2015). Use renewables to be cleaner: Meta-analysis of the renewable energy consumption–economic growth nexus. Renew. Sustain. Energy Rev..

[40] Rasul G., Sharma B. (2016). The nexus approach to water-energy-food security: An option for adaptation to climate change. Clim. Policy.

[41] Kaddoura S., Khatib S.E. (2017). Review of water-energy-food nexus tools to improve the nexus modelling approach for integrated policy making. Environ. Sci. Policy.

[42] Liu C.H., Cai W., Jia S., Zhang M.Y., Guo H.Y., Hu L.K., Jiang Z.G. (2018). Energy-based evaluation and improvement for sustainable manufacturing systems considering resource efficiency and environment performance. Energy Convers. Manag..

[43] Meng F., Liu G., Liang S., Su M., Yang Z. (2019). Critical review of the energy-water-carbon nexus in cities. Energy.

[44] Sherwood J. (2020). The significance of biomass in a circular economy. Bioresour. Technol..

[45] Chen H.Z., Xu J.F., Zhang K., Guo S.Z., Lv X., Mu X.Y., Yang L., Song Y., Hu X., Ma Y. (2022). New insights into the DPSIR model: Revealing the dynamic feedback mechanism and efficiency of ecological civilization construction in China. J. Clean. Prod..

[46] Zhuang X.Y., Xu Y.S., Zhang L., Li X., Lu J. (2022). Experiment and numerical investigation of inhalable particles and indoor environment with ventilation system. Energy Build..

[47] Sivek M., Jirásek J., Kavina P., Vojnarová M., Kurková T., Bašová A. (2020). Divorce after hundreds of years of marriage: Prospects for coal mining in the Czech Republic with regard to the European Union. Energy Policy.

[48] Gunzburger Y., Agnoletti M.F., Deshaies M., Ferey S., Raggi P. (2017). Social perception of unconventional gas extraction on the outskirts of a former coal-mining area in Northeast France. Extr. Ind. Soc..

[49] Trencher G., Downie C., Hasegawa K., Asuka J. (2020). Divestment trends in Japan’s international coal businesses. Renew. Sustain. Energy Rev..

[50] Xing M.L., Luo F.Z., Fang Y.H. (2021). Research on the sustainability promotion mechanisms of industries in China’s resource-based cities—From an ecological perspective. J. Clean. Prod..

[51] Wang M.Y., Li Y.M., Li J.Q., Wang Z.T. (2021). Green process innovation, green product innovation and its economic performance improvement paths: A survey and structural model. J. Environ. Manag..

[52] Li H.J., Long R.Y., Chen H. (2013). Economic transition policies in Chinese resource-based cities: An overview of government efforts. Energy Policy.

[53] Wu X., Zhang J.J., Geng X.L., Wang T., Wang K., Liu S.D. (2020). Increasing green infrastructure-based ecological resilience in urban systems: A perspective from locating ecological and disturbance sources in a resource-based city. Sustain. Cities Soc..

[54] Jing Z.R., Wang J.M., Tang Q., Liu B., Niu H.B. (2021). Evolution of land use in coal-based cities based on the ecological niche theory: A case study in Shuozhou City, China. Resour. Policy.

[55] Wang Y., Fang X.L., Yin S.W., Chen W. (2021). Low-carbon development quality of cities in China: Evaluation and obstacle analysis. Sustain. Cities Soc..

[56] Brown D., McGranahan G. (2016). The urban informal economy, local inclusion and achieving a global green transformation. Habitat Int..

[57] Guan X.L., Wei H.K., Lu S.S., Dai Q., Su H.J. (2018). Assessment on the urbanization strategy in China: Achievements, challenges and reflections. Habitat Int..

[58] Hardin G. (1968). The Tragedy of the Commons. Science.

[59] Pearce D. (1989). Blueprint for a Green Economy.

[60] Kaufmann R.K. (1993). Blueprint for a green economy: David Pearce, Anil Markandya and Edward B. Barbier. Earthscan, London, Great Britain, 1989. 192 pp. Ecol. Econ..

[61] Zhuang X.Y., Zhang L., Lu J. (2022). Past—Present—Future: Urban spatial succession and transition of rail transit station zones in Japan. Int. J. Environ. Res. Public Health..

[62] Cao M.X., Lu X.Y., Qin Z.X., Liu X.L., Fei L. (2022). Social-ecological system changes in China from 1990 to 2018. Ecol. Indic..

[63] Hu A.G., Zhou S.J. (2014). Green Development: Functional definition, mechanism analysis and development strategy. China Popul. Resour. Environ..

[64] Zeng X.G., Duan C.R. (2018). Study on performance evaluation and regional differences of green transformation in coal-resource-exhausted cities. China Popul. Resour. Environ..

[65] Liu X.W., Shen W.F., Duan P.X., Chen X.L. (2020). Evaluation of sustainable development transformation of coal-resource-exhausted cities in the new era. China Min. Mag..

[66] Tao X.Y., Wu Z.Y., Li X.F., Zhu J.L., Chu Y., Chang Y.M. (2022). Performance measurement and obstacle factor analysis of the transformation and development of coal resource- exhausted cities: A case study of Jiaozuo City. Ecol. Econ..

[67] Dwivedi P.P., Sharma D.K. (2022). Application of Shannon Entropy and COCOSO techniques to analyze performance of sustainable development goals: The case of the Indian Union Territories. Results Eng..

[68] Yu L.E. (2022). Evaluation of green transformation model of coal-resource-exhausted cities based on AHP. J. Zaozhuang Univ..

[69] Dewa D.D., Buchori I., Sejati A.W., Liu Y. (2022). Shannon Entropy-based urban spatial fragmentation to ensure sustainable development of the urban coastal city: A case study of Semarang, Indonesia. Remote Sens. Appl. Soc. Environ..

[70] Saiu V., Blečić I., Meloni I. (2022). Making sustainability development goals (SDGs) operational at suburban level: Potentials and limitations of neighborhood sustainability assessment tools. Environ. Impact Assess. Rev..

[71] Cunha-Zeri G., Guidolini J.F., Branco E.A., Ometto J.P. (2022). How sustainable is the nitrogen management in Brazil? A sustainability assessment using the Entropy Weight Method. J. Environ. Manag..

[72] Xiong X.Q., Ma Q.R., Yuan Y.Y., Wu Z.H., Zhang M. (2020). Current situation and key manufacturing considerations of green furniture in China: A review. J. Clean. Prod..

[73] Wei F.W. (2021). Toward post-2020 global biodiversity conservation: Footprint and direction in China. Innovation.

[74] Mihelcic J.R., Crittenden J.C., Small M.J., Shonnard D.R., Hokanson D.R., Zhang Q., Chen H., Sorby S.A., James V.U., Sutherland J.W. (2003). Sustainability science and engineering: The emergence of a new metadiscipline. Environ. Sci. Technol..

[75] Zhang C., Miao C.H., Zhang W.Z., Chen X.H. (2018). Spatiotemporal patterns of urban sprawl and its relationship with economic development in China during 1990–2010. Habitat Int..

[76] Voorspools K. (2004). Sustainability of the future: Rethinking the fundamentals of energy research. Renew. Sustain. Energy Rev..

[77] National Bureau Statistics of China (2020). China Statistical Yearbook.

[78] Pandey B., Agrawal M., Singh S. (2014). Assessment of air pollution around coal mining area: Emphasizing on spatial distributions, seasonal variations and heavy metals, using cluster and principal component analysis. Atmos. Pollut. Res..

[79] Lloyd S., Mohseni M., Rebentrost P. (2014). Quantum principal component analysis. Nat. Phys..

[80] Jing Z., Wang J. (2020). Sustainable development evaluation of the society-economy-environment in a resource-based city of China: A complex network approach. J. Clean. Prod..

[81] Xu Y.S., Zhuang X.Y. (2022). Container shipping scheduling method based on the evidence reasoning approach in fluctuating CCFI and BDI cycle. Math. Probl. Eng..

[82] Destoumieux-Garzón D., Matthies-Wiesler F., Bierne N., Binot A., Boissier J., Devouge A., Garric J., Gruetzmacher K., Grunau C., Guégan J.F. (2022). Getting out of crises: Environmental, social-ecological and evolutionary research is needed to avoid future risks of pandemics. Environ. Int..

[83] Jalaei F., Guest G., Gaur A., Zhang J.Y. (2020). Exploring the effects that a non-stationary climate and dynamic electricity grid mix has on whole building life cycle assessment: A multi-city comparison. Sustain. Cities Soc..

[84] Ghose A., McLaren S.J., Dowdell D. (2020). Upgrading New Zealand’s existing office buildings—An assessment of life cycle impacts and its influence on 2050 climate change mitigation target. Sustain. Cities Soc..

[85] Rayan M., Gruehn D., Khayyam U. (2022). Planning for Sustainable Green Urbanism: An Empirical Bottom-Up (Community-Led) Perspective on Green Infrastructure (GI) Indicators in Khyber Pakhtunkhwa (KP), Pakistan. Int. J. Environ. Res. Public Health.

[86] Zameer H., Muhammad S., Xuan V.V. (2020). Reinforcing poverty alleviation efficiency through technological innovation, globalization, and financial development. Technol. Forecast. Soc. Chang..

[87] Hong Q.Q., Cui L.H., Hong P.H. (2022). The impact of carbon emissions trading on energy efficiency: Evidence from quasi-experiment in China’s carbon emissions trading pilot. Energy Econ..

[88] Li Z., Cai Y.P., Lin G. (2022). Pathways for sustainable municipal energy systems transition: A case study of Tangshan, a resource-based city in China. J. Clean. Prod..

[89] Santos M., Moreira H., Cabral J.A., Gabriel R., Teixeira A., Bastos R., Aires A. (2022). contribution of home gardens to sustainable development: Perspectives from a supported opinion essay. Int. J. Environ. Res. Public Health.

[90] Liu T., Ren C., Zhang S., Yin A., Yue W. (2022). Coupling coordination analysis of urban development and ecological environment in yrban Area of Guilin based on multi-source data. Int. J. Environ. Res. Public Health.

[91] Zhang C., Li J., Liu T., Xu M., Wang H., Li X. (2022). The spatiotemporal evolution and influencing factors of the Chinese cities’ ecological welfare performance. Int. J. Environ. Res. Public Health.

[92] Karimi N., Tsun K., Ng W., Richter A. (2022). Development of a regional solid waste management framework and its application to a prairie province in central Canada. Sustain. Cities Soc..

[93] Abubakar I.R., Maniruzzaman K.M., Dano U.L., AlShihri F.S., AlShammari M.S., Ahmed S.M.S., Al-Gehlani W.A.G., Alrawaf T.I. (2022). Environmental Sustainability Impacts of Solid Waste Management Practices in the Global South. Int. J. Environ. Res. Public Health.

[94] Harijani A.M., Mansour S. (2022). Municipal solid waste recycling network with sustainability and supply uncertainty considerations. Sustain. Cities Soc..

[95] Rockström J. (2016). Future Earth. Science.

